# Pronounced cancer resistance in a subterranean rodent, the blind mole-rat, *Spalax*: *in vivo* and *in vitro* evidence

**DOI:** 10.1186/1741-7007-11-91

**Published:** 2013-08-09

**Authors:** Irena Manov, Mark Hirsh, Theodore C Iancu, Assaf Malik, Nick Sotnichenko, Mark Band, Aaron Avivi, Imad Shams

**Affiliations:** 1 Institute of Evolution, University of Haifa, Haifa 31095, Israel; 2 Department of Pathology, Assaf Harofeh Medical Center, Zerifin 70300, Israel; 3 Milman-David Foundation for Pediatric Research, Haifa 34355, Israel; 4 Veterinary Services, Research Authority, University of Haifa, Haifa 31905, Israel; 5 The W.M. Keck Center for Comparative and Functional Genomics, University of Illinois Urbana-Champaign, Urbana, IL 61801, USA

## Abstract

**Background:**

Subterranean blind mole rats *(Spalax)* are hypoxia tolerant (down to 3% O_2_), long lived (>20 years) rodents showing no clear signs of aging or aging related disorders. In 50 years of *Spalax* research, spontaneous tumors have never been recorded among thousands of individuals*.* Here we addressed the questions of (1) whether *Spalax* is resistant to chemically-induced tumorigenesis, and (2) whether normal fibroblasts isolated from *Spalax* possess tumor-suppressive activity.

**Results:**

Treating animals with 3-Methylcholantrene (3MCA) and 7,12-Dimethylbenz(a) anthracene/12-*O*-tetradecanoylphorbol-13-acetate (DMBA/TPA), two potent carcinogens, confirmed *Spalax* high resistance to chemically induced cancers. While all mice and rats developed the expected tumors following treatment with both carcinogens, among *Spalax* no tumors were observed after DMBA/TPA treatment, while 3MCA induced benign fibroblastic proliferation in 2 *Spalax* individuals out of12, and only a single animal from the advanced age group developed malignancy 18 months post-treatment. The remaining animals are still healthy 30 months post-treatment. *In vitro* experiments showed an extraordinary ability of normal *Spalax* cultured fibroblasts to restrict malignant behavior in a broad spectrum of human-derived and in newly isolated *Spalax* 3MCA-induced cancer cell lines. Growth of cancer cells was inhibited by either direct interaction with *Spalax* fibroblasts or with soluble factors released into culture media and soft agar. This was accompanied by decreased cancer cell viability, reduced colony formation in soft agar, disturbed cell cycle progression, chromatin condensation and mitochondrial fragmentation. Cells from another cancer resistant subterranean mammal, the naked mole rat, were also tested for direct effect on cancer cells and, similar to *Spalax*, demonstrated anti-cancer activity. No effect on cancer cells was observed using fibroblasts from mouse, rat or *Acomys*. *Spalax* fibroblast conditioned media had no effect on proliferation of noncancerous cells.

**Conclusions:**

This report provides pioneering evidence that *Spalax* is not only resistant to spontaneous cancer but also to experimentally induced cancer, and shows the unique ability of *Spalax* normal fibroblasts to inhibit growth and kill cancer cells, but not normal cells, either through direct fibroblast-cancer cell interaction or via soluble factors. Obviously, along with adaptation to hypoxia, *Spalax* has evolved efficient anti-cancer mechanisms yet to be elucidated. Exploring the molecular mechanisms allowing *Spalax* to survive in extreme environments and to escape cancer as well as to kill homologous and heterologous cancer cells may hold the key for understanding the molecular nature of host resistance to cancer and identify new anti-cancer strategies for treating humans.

## Background

Throughout the last 50 years, several thousand *Spalax* individuals have been housed and studied in the Animal Facility at the Institute of Evolution of Haifa University. Despite this small rodent’s (approximately 100 to 200 gr.) long lifespan (>20 years), none of the animals have ever developed spontaneous tumors, nor do they show any aging-related phenotypic changes. The mole rat, *Spalax ehrenbergi,* is a wild, solitary rodent of the Eastern Mediterranean region. *Spalax* inhabits a system of poorly ventilated, dark, sealed underground tunnels protected from climatic extremes, pathogens and predation. During the Mediterranean rainy season animals are engaged in intensive digging to collect food, mate, and repair and extend their territory under extreme hypoxic conditions. *Spalax* has evolved a unique adaptive complex mechanism for surviving underground, including a special ability to cope with extreme hypoxia and hypercapnia [1]. *Spalax* can conduct intensive aerobic work under low O_2_ pressures (down to 3% O_2_) due to increased muscular mass, and high density of blood vessels and mitochondria, resulting in reduced oxygen diffusion distance and efficient oxygen delivery even at low capillary PO_2_[1,2].

Hypoxia can result in a failure to maintain essential cellular functions and contributes to cardio- and cerebrovascular failure, pulmonary diseases and cancer, which together are the primary sources of morbidity in the Western world. A long and growing list of genes exhibits hypoxia-related adaptations in structure and function in *Spalax*[3-6]. Noteworthy are *VEGF*, constitutively highly expressed as compared to rats [7]; *p53* that harbors substitutions in the DNA-binding site, identical to the most common *p53* mutations in tumors; however, in *Spalax* it renders a bias against apoptosis but favors cell cycle arrest/DNA repair both *in vitro* and *in vivo*[8]; and a unique *Spalax* heparanase splice variant that was shown to decrease tumor size in mice by a factor of 7 and reduce metastatic activity compared to native mice heparanase [9]. Furthermore, assessment of *Spalax* transcriptome assembly and expression data has revealed enrichment of genes that overlap cancer resistance, apoptosis, angiogenesis pathways and hypoxia-tolerance [10,11]. This suggests that *Spalax* is potentially resistant to malignant transformation. Elucidating the mechanisms evolved in this wild, non-inbred, naturally cancer resistant rodent should have great importance as preventative measures and may present an efficient way of dealing with increasing cancer incidence.

Tumors contain malignant cells and tumor stroma consisting of fibroblasts, extracellular matrix (ECM) and vasculature with endothelial cells [12,13]. Cancer progression requires a permissive stromal environment in which mutant cells can survive, proliferate and invade. Fibroblasts are ubiquitous stromal cells interlinked with tumors via regulation of growth factors and cytokines, and through reassembling of the ECM [14]. The majority of published studies report the cancer-enhancing effects of fibroblasts in their activated form [15,16]. However, early studies from co-culture experiments indicate that normal fibroblasts may have a tumor suppressor function [16]. Unfortunately, little attention has been given to the protective role of normal fibroblasts.

Based on our earlier observations that *Spalax* is resistant to spontaneous cancer, and assuming that normal fibroblasts apparently play a role in this phenomenon, we took two experimental approaches in the present study: (1) to directly confirm the hypothesis that *Spalax* is highly resistant to induced tumorigenesis, we used a two-step 7,12-Dimethylbenz (a) anthracene/12-*O*-tetradecanoylphorbol-13-acetate (DMBA/TPA) skin carcinogenesis protocol [17], and 3-Methylcholantrene (3MCA) protocol for local fibrosarcoma induction [18] in mice, rats and *Spalax*; and (2) co-culture experiments were conducted to study the interactions between normal primary fibroblasts isolated from different rodent species (*Spalax*, mouse, rat, naked mole rat *Heterocephalus glaber* and spiny mice *Acomys cahirinus*), with human hepatocellular carcinoma (Hep3B and HepG2) and breast cancer cells (MDA-MB-231 and MCF7), as well as 3MCA-induced, *Spalax*-derived fibrosarcoma cells (*Sp*FS2240).

We provide evidence that (1) *Spalax* is extremely resistant to experimentally induced cancer, and (2) *Spalax’s* normal fibroblasts, originated from adult or newborn animals, target tumor cells and restrict malignant behavior either through direct fibroblast-cancer cell interaction or via soluble factors produced by a monolayer of *Spalax* fibroblasts.

## Results

### *Spalax* is resistant to chemically-induced cancer

To assess experimentally if *Spalax* is resistant to chemically-induced carcinogenesis, we treated animals from different rodent species according to the following protocols:

#### DMBA/TPA treatment

**Figure 1:**
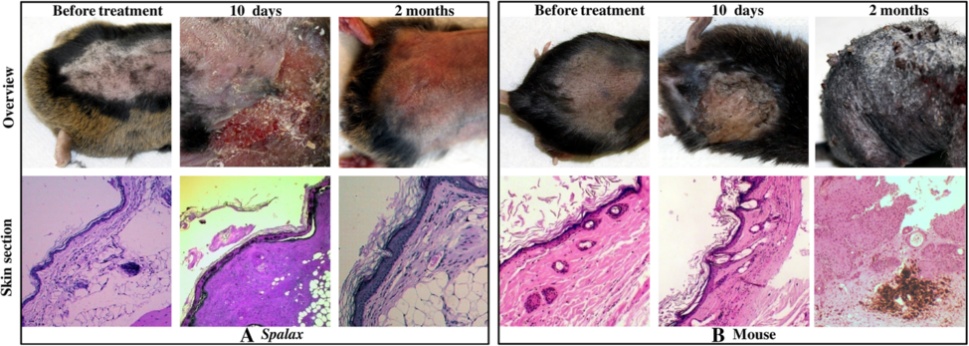
**Effect of DMBA/TPA carcinogenic applications on *Spalax *and mice skin*.*** Macroscopic and microscopic skin changes in *Spalax***(A)** and mice **(B)**. **(A)** Normal tissues (left images). Necrosis of skin and subcutaneous adipose tissue (middle images). Completely healed skin lesion showing epidermal thickening with hyperkeratosis and dermal fibrosis (right images). Hematoxylin and eosin staining, ×40 (left and middle images) and ×100 (right image). **(B)** Normal tissues (left images). Intra-epidermal blisters, partially ruptured with erosion formation and crusting, congestion and inflammatory cell infiltrate within the dermis indicate ongoing inflammation (middle images). Skin papillary outgrowths with thickened, dysplastic epidermis, numerous mitoses and foci are suggestive of squamous cell carcinoma (right image). Hematoxylin and eosin staining, ×40 (left and middle images) and ×100 (right image). DMBA/TPA, 7,12-Dimethylbenz(a) anthracene/12-*O*-tetradecanoylphorbol-13-acetate.

*Spalax* and C57BL/6 mice were treated with DMBA/TPA to induce skin cancer [19]. *Spalax* animals developed skin lesions within 10 days (Figure 1A, upper middle panel). Histological examination of hematoxylin and eosin-stained tissue sections demonstrated skin necrosis involving the deep parts of the dermis, massive infiltration of the affected areas with neutrophil leukocytes, and ulcerated epidermis focally covered with fibrino-purulent exudates (Figure 1A, lower middle panel). The subcutaneous skeletal muscle and bone tissues were not affected, and no tumor was identified. The wounds completely healed within seven to nine weeks, resulting in epidermal thickening (Figure 1A, right panels), and no further progression to skin tumors was observed, even though TPA treatments were extended to six months (November 2010 to April 2011). In the control group, *Spalax* animals treated with acetone only did not show any changes in their skin macro- and microstructure, similar to non-treated animals (Figure 1A, left panels). Following 7 to 10 days of DMBA/TPA treatment, mice demonstrated small intra-epidermal blisters; some of them ruptured, forming superficial erosions with extensive crusting (Figure 1B, middle panels), which subsequently underwent transformation into multiple skin tumors within two to three months (Figure 1B, upper right panel). Histological examination revealed papillary and flat epidermal outgrowths with dysplastic features, focally similar to squamous cell carcinoma (Figure 1B, right panels).

#### 3-MCA treatment

**Figure 2:**
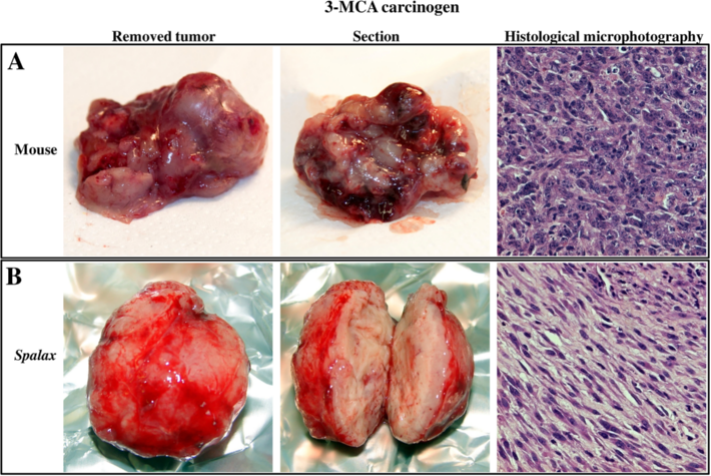
**Effect of 3-Methylcholantren treatment on soft tissue tumor induction in *Spalax *and mice.** Animals treated with a single injection of 3MCA as described in the Materials and methods section. Representative images show macroscopic and microscopic observations. Mice **(A)**: An ill-defined, soft mass, with foci of necrosis and hemorrhage; diagnosed as high-grade fibrosarcoma by histology. *Spalax ***(B)**: a well-circumscribed, firm, whitish nodule composed of benign spindle cells organized into long regular bundles - benign reactive fibrosis. Hematoxylin and eosin staining, ×100. 3MCA, 3-Methylcholantrene.

The ability of a single subcutaneous 3-MCA injection to induce fibrosarcoma is well documented [20]. The expected tumors appeared within two to three months in mice, and in four to six months in rats. Hypercellular spindle cell tumors with highly pleiomorphic, extensively proliferating cells (30 and more mitotic figures per 10 high power fields) arranged into intersected bundles or wide sheets were identified. Scant, partially myxoid stroma and areas of hemorrhagic necrosis were typical findings (Figure 2A). All examined tumors developed in 3-MCA-treated mice and rats were histologically identified as fibrosarcomas. Importantly, *Spalax* did not show any pathological process for over a year. However, by 14 to 16 months following the 3-MCA treatment, 2 of the *Spalax* animals (out of 6 old individuals and a total of 12 animals) developed a tissue overgrowth at the site of the injection. These lesions were well circumscribed in shape, unlike the ill-defined tumors found in mice and rats (Figure 2B). Histological examination revealed benign spindle cell proliferation most probably reflecting fibrosis at the site of an incompletely resolved inflammatory reaction.

### A case of fibrosarcoma development in *Spalax*

**Figure 3:**
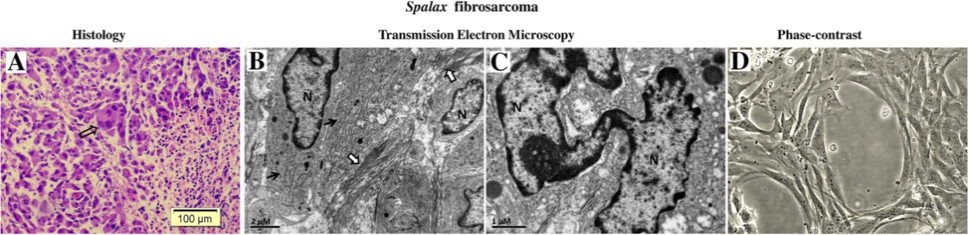
**3MCA-induced tumor in *Spalax*. (A)** Light microscopic examination. Note spindle, epithelioid and giant multinuclear cells (empty arrow); nuclei are variable in shape, size and chromatin distribution; nucleoli vary in frequency. Hematoxylin and eosin staining, ×100. **(B)** Transmission electron microscopy (TEM): dilated, elongated rough endoplasmic reticulum (black arrows) and abundant collagen fibers (white arrows) **(C)** TEM: a giant, monstrous nucleus (N). **(D)** Cell line established from *Spalax* tumor, phase contrast image after six months of continuous cultivation (*×*200). 3MCA, 3-Methylcholantrene.

A single, old *Spalax* individual developed a 3-MCA-induced tumor 18 months after initial treatment (Figure 3). A biopsy was performed, and the histological examination revealed a partially necrotic and heavily inflamed, spindle and epithelioid cell tumor with infiltrative borders and myxoid stroma. Cells demonstrated dyscohesion, polymorphism in size and shape (bizarre and giant cells present) and prominent nuclear atypia (Figure 3A). This hypercellular tumor demonstrated high mitotic activity (above 30 mitoses per 10 high power fields) with abundant atypical mitotic figures. Transmission electron microscopy revealed fibrosarcoma-like findings [21]: deformed nuclei, some with monstrous appearance; long branching and dilated rough endoplasmic reticulum and abundance of extracellular collagen fibers (Figure 3B,C). Myofibroblastic differentiation features were not observed. An immortal cell line was established from the tumor sample. The cultured adherent cells show a typical fibroblast phenotype (Figure 3D), which has remained unchanged throughout a long culture time (40 passages, 8 months after isolation).

The remaining treated *Spalax* individuals showed no phenotypic or behavioral changes, and were still under observation in the Animal House over two years following treatment (October 2010 to July 2013).

### *Spalax* fibroblasts suppress growth of human cancer cells *in vitro*

**Figure 4:**
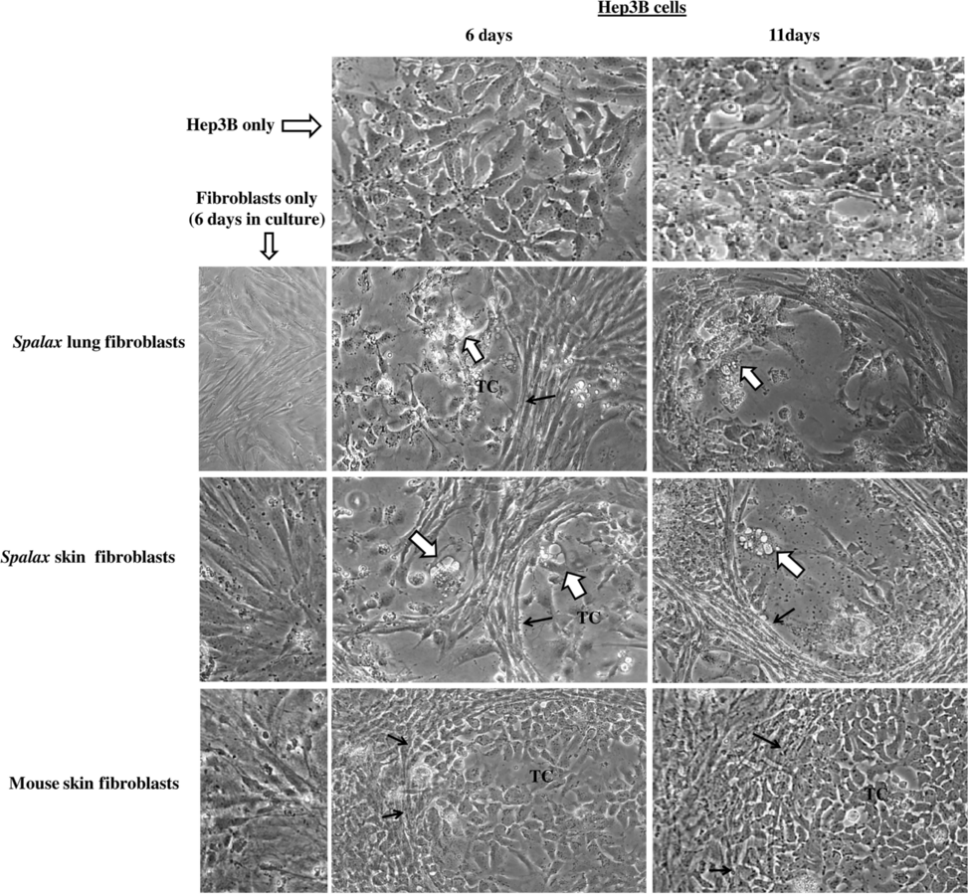
**Effects of *Spalax *and mouse fibroblasts on growth of co-cultured human hepatoma cells.** Tumor cells (TC) were cultured either alone or in the presence of *Spalax* or mouse fibroblasts in the ratio of 1:10 (5 × 10^4^ fibroblasts and 5 × 10^3^ cancer cells in six-well plates) in RPMI/DMEM-F12 media (1:1) containing 10% FBS. White arrows point to the foci of destroyed cancer cells, and black arrows show the fibroblast-tumor cell colony boundaries. Cells in mono- and co-cultures were observed and photographed daily. Representative images for each sample at different time intervals are shown (×200).

**Figure 5:**
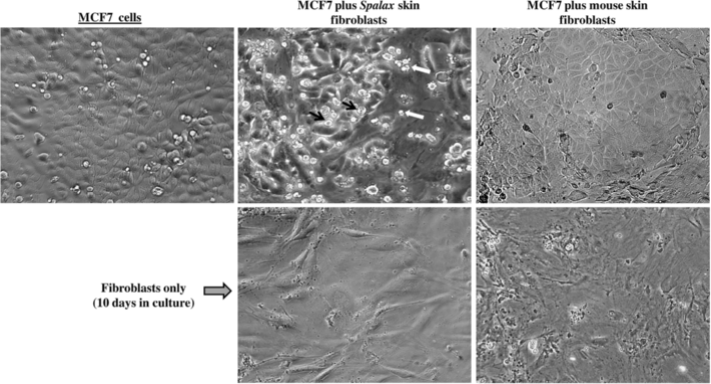
**Morphologic alterations in human breast cancer MCF7 cells triggered by co-culture with *Spalax *fibroblasts.** MCF7 cells were co-cultured with skin fibroblasts of *Spalax* or mouse in the ratio of 1:15 (5 × 10^4^ fibroblasts and 2.5 × 10^3^ cancer cells in six-well plates) in DMEM/DMEM-F12 media (1:1) containing 5% FBS. Representative phase contrast images after 10 days of co-culture are presented (×200). Note rounding and detachment of MCF7 cells co-cultured with *Spalax* fibroblasts*.* Black arrows point to rounding cells. White arrows show shrunken “floating” cells.

To compare the effects of normal fibroblasts isolated from different rodents on the growth of human cancer cells, we used a co-culture approach, where fibroblasts were cultured together with cancer cells on a shared surface (Figures 4 and 5). In these experiments, hepatoma-derived Hep3B cells as well as breast cancer MCF7 cells were tested. Obvious inhibition of cancer cell growth was found when Hep3B cells were co-cultured with *Spalax* normal lung and skin fibroblasts: the foci of destroyed cancer cells were visible after six days of co-culture (Figure 4). Prolonged co-cultivation up to 11 days resulted in further destruction of cancer cell colonies by the presence of *Spalax* fibroblasts and the spaces previously occupied by Hep3B cells were invaded by fibroblasts (Figure 4). In contrast, the number of cancer cells co-cultured with mouse fibroblasts increased gradually, and on Day 6, Hep3B cells surrounded by mouse fibroblasts reached approximately 80% confluence, similar to control (Hep3B only). Overgrown Hep3B colonies were found after 11-day co-culture with mouse fibroblasts. An obvious inhibitory effect was demonstrated when *Spalax* normal skin fibroblasts were co-cultured with breast cancer MCF7 cells as well (Figure 5). After 10 days of co-culture with *Spalax* fibroblasts, massive rounding and detachment of cancer cells were observed. On the other hand, mouse fibroblasts stimulated proliferation of MCF7 cells, and by Day 10 densely populated colonies of cancer cells developed.

### *In vitro* anticancer activity by other wild, natural rodents’ fibroblasts

**Figure 6:**
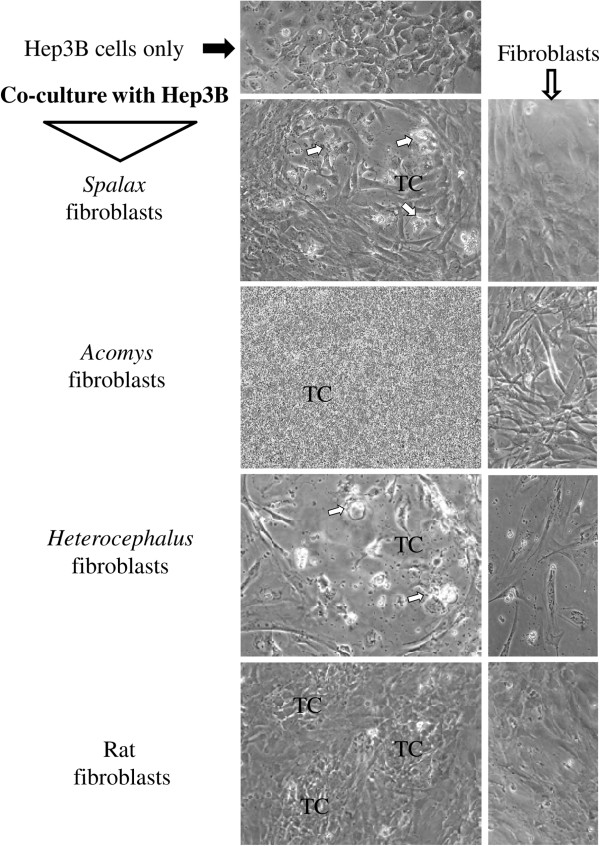
**Comparison of the effects of *Spalax, Acomys, Heterocephalus *and rat skin fibroblasts on growth of Hep3B cells.** Hep3B tumor cells were cultured either alone or in presence of *Spalax*, *Acomys*, *Heterocephalus* or rat fibroblasts in the ratio of 1:10 (5 × 10^4^ fibroblasts and 5 × 10^3^ cancer cells in six-well plates) in RPMI/DMEM-F12 media (1:1) containing 10% FBS. After seven days incubation cells were photographed. Representative images for each sample are shown (×200). White arrows point to the foci of damaged cancer cells. TC, tumor cells.

Since we compare a wild mammal with laboratory animals that are sensitive to cancer, we conducted co-culture experiments using Hep3B cancer cells with skin fibroblasts isolated from two different wild, natural rodents: *Acomys*, a short-lived, wild, above-ground rodent; and naked mole rat (*Heterocephalus glaber*), a long-lived cancer-resistant wild subterranean rodent [22]. As shown (Figure 6), no growth inhibitory effect was found when *Acomys* fibroblasts were co-cultured with Hep3B cells. On the contrary, *Acomys* fibroblasts promoted cancer cell invasion similar to the effect of rat fibroblasts. *Heterocephalus* cells, similar to *Spalax*, evidently destroyed cancer cell growth (Figure 6).

### Conditioned medium generated by *Spalax* fibroblasts induces cancer cell death, but does not affect normal primary fibroblasts

**Figure 7:**
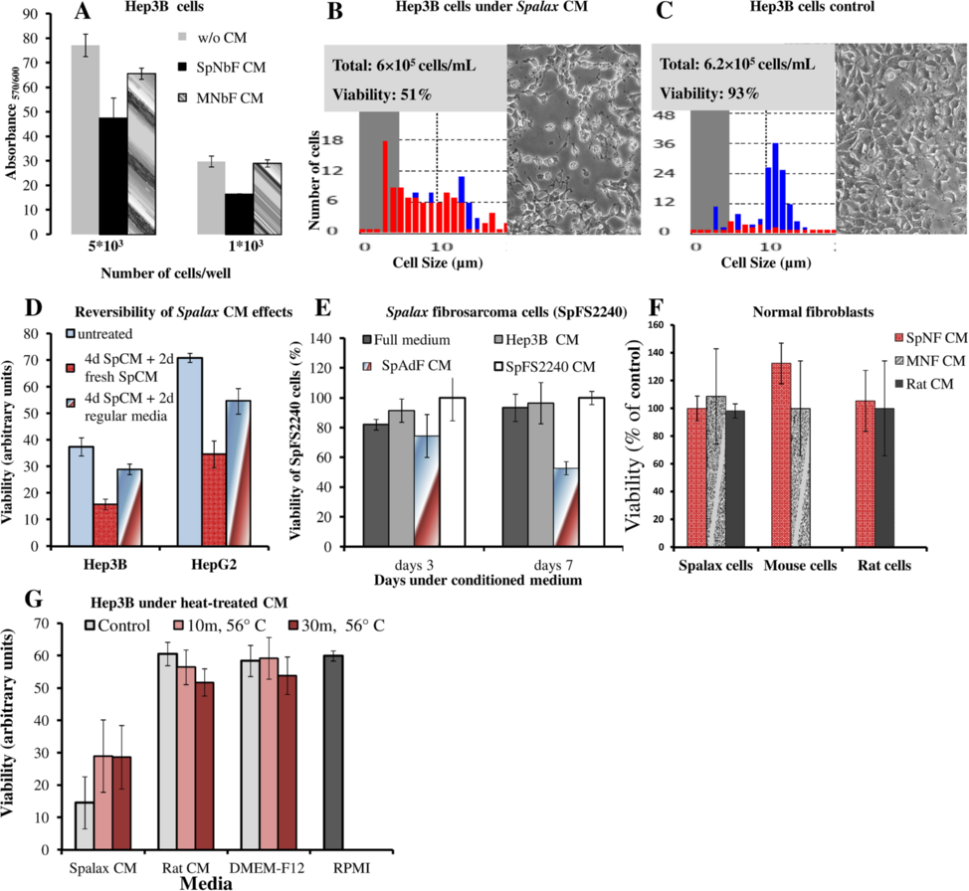
**Effects of conditioned media (CM) on viability of cancerous and non-cancerous cells. (A)** Hep3B cells were seeded in a 96-well plate at a density of 5 × 10^3^ and 1 × 10^3^ cells/well in RPMI-DMEM/F12 medium conditioned by *Spalax* or mouse skin newborn fibroblasts (*Sp*NbF and MNbF, respectively). Hep3B cells were incubated for four days; viability was estimated by PrestoBlue® Reagent. **(B,C)** Hep3B cells (1 × 10^4^ cell/well) were cultured in six-well plates under conditioned medium of *Spalax* adult skin fibroblasts **(B)** or grown in medium generated by Hep3B cells **(C)**. After nine days, the cells’ survival rates were assessed by a Countess® cell counter (Life Technologies); red: dead cells, blue: viable cells. **(D)** Hep3B and HepG2 cells were incubated under *Spalax* CM for four days, followed by changing the media either to fresh media or new *Spalax* CM. After two days, viability was estimated by PrestoBlue® Reagent. **(E)***Spalax* fibrosarcoma cells (*Sp*FS2240) were incubated for three or seven days in full medium or under CM of *Spalax* adult skin normal fibroblasts (*Sp*AdF CM), Hep3B (Hep3B CM), *Spalax* fibrosarcoma (*Sp*FS2240 CM). Cell viability was evaluated by using PrestoBlue® reagent. Results are presented as percentage of control (*Sp*FS2240 CM); mean ± S.D. **(F)** Effects of CM generated by *Spalax* or mouse normal fibroblasts (*Sp*NbF CM and MNbF CM, respectively) on the growth of non-cancerous cells. The viability was estimated after four days by PrestoBlue® reagent; mean ± S.D. **(G)** Heat treatment of conditioned media. Seven-day CM, generated by *Spalax* or rat fibroblasts, was heat-treated at 56°C for 10 minutes and 30 minutes prior to addition to Hep3B cancer cells (2,000 cell/well) in 96-well plates. Cells were incubated for seven days followed by PrestoBlue® test. All results were obtained from three independent experiments performed in three to six technical repeats.

To determine whether the anti-cancer activity of *Spalax* fibroblasts was mediated by fibroblast-secreted soluble factors, conditioned media (CM) obtained from *Spalax*, mouse and rat monolayers were tested. Cancer cells of different origins were incubated under CM of normal fibroblasts, which had never been exposed to cancer cells or other stimuli. Effects of CM generated by cancer cells were also tested (Figure 7). As demonstrated in Figure 7A, exposure of Hep3B cells to CM from cultured newborn *Spalax* fibroblasts decreased cancer cell viability as measured by mitochondrial respiratory function. Exposure to mouse CM hardly had an effect on cancer cell viability. Similarly, nine-day exposure of Hep3B cells to CM generated by adult (>5.5 years old) *Spalax* fibroblasts obviously reduced cancer cell viability as was determined by a trypan blue extrusion assay (Figure 7B,C): cancer cells exposed to *Spalax* fibroblast-conditioned CM reached 49% death, whereas unexposed cells remained completely adherent and viable (Figure 7C).

We next evaluated the reversibility of the inhibition of cancer cells initiated by *Spalax* CM. HepG2 and Hep3B were grown with *Spalax* CM for four days, then the medium was changed by either fresh unused regular media or with fresh *Spalax* CM. Cancer cell viability was measured after another two days. Recovery of the cancer cells was demonstrated when the CM was changed with fresh unused regular media (Figure 7D). Importantly, growth of *Spalax-*derived fibrosarcoma cells (*Sp*FS2240) was gradually suppressed by CM generated by *Spalax* normal fibroblasts, but was not affected by normal, full medium and CM derived from Hep3B cells or CM derived from the *Sp*FS2240 cells themselves (Figure 7E). Noteworthy, no inhibitory effects were detected on mouse, rat and *Spalax* normal fibroblasts following exposure to homologous or heterologous CM (Figure 7F). To get a preliminary idea of the nature of the secreted factors responsible for cancer cell growth inhibition, CM from *Spalax* and rat fibroblasts, and the regular medium of fibroblasts (DMEM-F12) were heated to 56°C for 10 minutes, and 30 minutes. The different heat-treated media was mixed 1:1 with RPMI (the optimal growth medium for the hepatoma cell lines used in this study) and was added to Hep3B cancer cells. After seven days, the viability of the cancer cells was measured. The heat-treated CM generated from *Spalax* fibroblasts reduced its anticancer activity, expressed as a partial increase in Hep3B cells viability (Figure 7G).

### Soluble factors generated by *Spalax* fibroblasts cause cell cycle arrest, nuclear fragmentation, and impair mitochondrial dynamics in cancer cells

**Figure 8:**
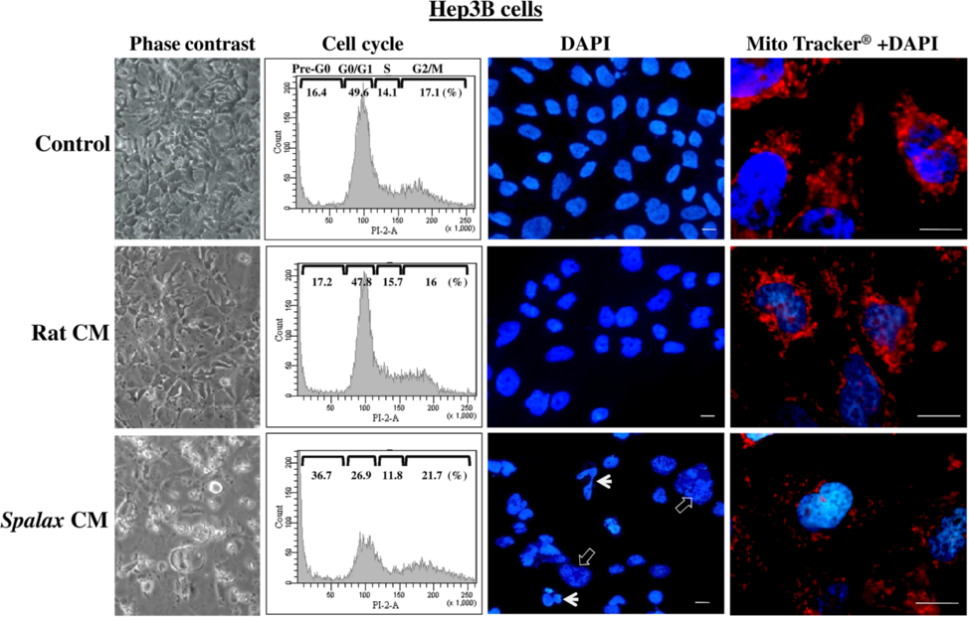
***Spalax *fibroblast-conditioned medium compromises cell cycle, causes nuclear and mitochondrial fragmentation in Hep3B cells.** Hep3B cells were grown on cover slips under medium conditioned by *Spalax* or rat fibroblasts for seven days. Representative phase-contrast images demonstrating morphological changes (×200) are depicted. Cells were harvested and stained with PI, and cell cycles were analyzed by flow cytometry. Representative flow cytometry histograms of three independent experiments performed in duplicate are presented. Hep3B cells were stained with MitoTracker®Red, fixed with formaldehyde and counterstained with DAPI. Representative fluorescence microscopy images demonstrating nuclear and mitochondrial changes are present. White arrows point fragmented nuclei; empty arrows point chromatin condensation. Scale bars represent 10 μm. PI, Propidium iodide.

**Figure 9:**
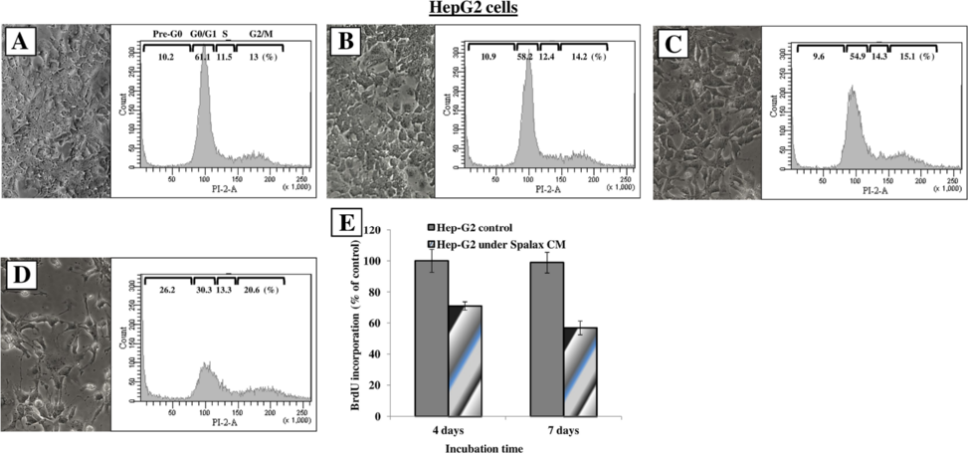
**Effects of *Spalax*, mouse and rat conditioned media on morphology and cell cycle progression in HepG2 cells.** HepG2 cells were incubated under conditioned media for eight days; thereafter, cell morphology was documented using phase contrast microscopy, harvested, stained with PI and analyzed by flow cytometry. Representative images (×200) and flow cytometry histograms are presented: **(A)** control media; **(B)** rat CM; **(C)** mouse CM; **(D) ***Spalax* CM; **(E)** BrdU incorporation assay: HepG2 were grown in 96-well plates (2000 cells/well) for four and seven days under *Spalax*-generated CM. BrdU Cell Proliferation ELISA (Exalpha) was used. Time-dependent decrease in cell proliferation under *Spalax*-generated CM is depicted. CM, Conditioned media; PI, Propidium iodide.

To investigate the mechanisms by which *Spalax* fibroblasts induce cancer cell death, we examined nuclear and mitochondrial shape dynamics, as well as cell cycle distributions in Hep3B and HepG2 cells. No changes in the morphology of cells, nuclei and mitochondria as well as in cell cycle distribution were found when Hep3B cells were incubated with rat CM (Figure 8, middle row) compared to Hep3B grown with their own medium (Figure 8, upper row; control). In contrast, following exposure to *Spalax* CM, Hep3B cells undergo phenotypic changes observed under phase contrast microscopy: cellular shrinkage, irregularities in the plasma membrane and blebs formation (Figure 8, lower row, phase-contrast). Cell cycle analysis revealed a noticeable accumulation of dead cells in sub-G1 (36.7% versus 16.4% in control), a reduction in the number of cells in G0/G1 (28.9% versus 49.6% in control), and a modest arrest of proliferation in G2/M (21.7% versus 17.1% in control) (Figure 8, lower row, cell cycle). Nuclear staining with DAPI of Hep3B cells that were grown with *Spalax* CM for eight days, revealed heterogeneous chromatin appearance within irregularly shaped nuclei, and in many cells extensive chromatin condensation and nuclear fragmentation were conspicuous (Figure 8, lower row, DAPI staining). On the other hand, homogeneous patterns with regular-shaped nuclei were mainly represented in the cells incubated with rat CM as well as in the control cells (Figure 8, upper and middle row, DAPI staining). To examine whether *Spalax* fibroblast CM could induce mitochondrial dynamic changes in cancer cells, Hep3B cells were stained with MitoTracker-Red® probe after eight days of incubation. Compared with control and rat CM, the mitochondrial network of cells after eight-day growth with *Spalax* CM demonstrated the presence of damaged fragmented mitochondria (Figure 8, lower row, MitoTracker® + DAPI). Similar to Hep3B cells, HepG2 cells under *Spalax* CM also showed morphological changes and accumulation of cells in sub-G0/G1 whereas mouse and rat CM did not affect cellular morphology and cell cycle distribution (Figure 9). BrdU incorporation into DNA, a marker for cell proliferation, confirmed a time-dependent anti proliferative effect of *Spalax* CM on HepG2 cancer cells (Figure 9E).

### *Spalax* normal fibroblasts inhibit colony formation in soft agar of the breast carcinoma cell lines MDA-MB-231 and MCF7 as well as *Spalax*-derived fibrosarcoma

**Figure 10:**
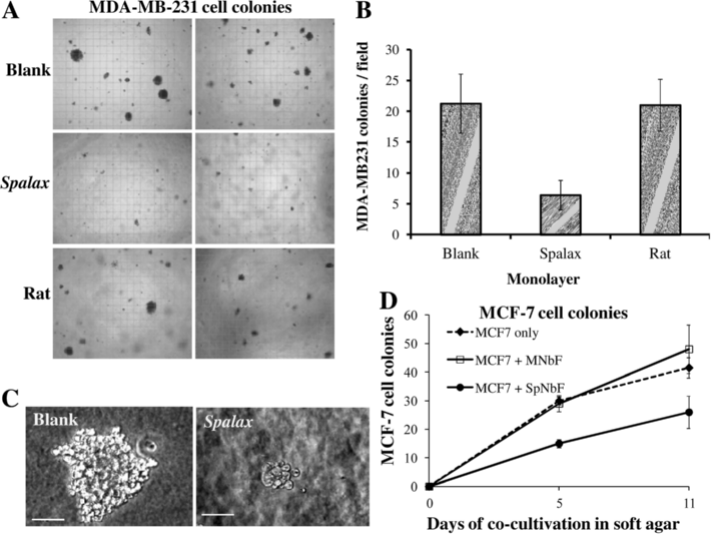
***Spalax *fibroblasts suppress colony formation of human breast cancer cells MDA-MB-231 and MCF7 in soft agar. (A)** MDA-MB-231 cells (5 × 10^3^ cells) *cells* were suspended in 0.35% agar and added as the cancer cell top layer to base layer either empty (blank) or containing the *Spalax* or rat fibroblast monolayer. At Day 21, colonies larger than 50 μm were counted under an inverted microscope and photographed (×40). Representative microscopic images out of 15 fields are shown. **(B)** Average number of colonies counted in soft agar (n = 15). The experiment was performed in duplicate plates at least three times; mean ± S.D. **(C)** A representative colony in soft agar was formed by MDA-MB-231 only, or by co-culturing with a *Spalax* fibroblast monolayer. The size bar shows equivalent magnification in both images (× 200). **(D)** MCF7 cells (5 × 10^3^ cells) were grown in soft agar on top of a monolayer of mouse newborn (MNbF), or *Spalax* newborn fibroblasts (*Sp*NbF) in 35-mm culture dishes. After 5 and 11 days of incubation colonies containing >20 cells were counted by using an inverted microscope (× 200), mean ± S.D.

To study whether soluble factors generated by *Spalax* fibroblasts may influence colony formation in soft agar, breast cancer cells were cultivated for three weeks in the absence or presence of *Spalax* fibroblasts (Figure 10). *Spalax* fibroblasts strongly reduced the formation of MDA-MB-231 colonies (Figure 10A,B). The ability of MDA-MB-231 to form large colonies was completely inhibited by *Spalax* fibroblasts (Figure 10C), while rat fibroblasts had no effect on colony formation (Figure 10A,B). Cells from another human breast cancer cell line, MCF-7, were incubated with monolayers of *Spalax* and mouse fibroblasts (Figure 10D). Remarkably, after 11 days, and compared to the control, more colonies were formed when human MCF7 cells were co-cultured with mouse fibroblasts, whereas a monolayer of *Spalax* fibroblasts significantly reduced MCF7 colony-formation.

**Figure 11:**
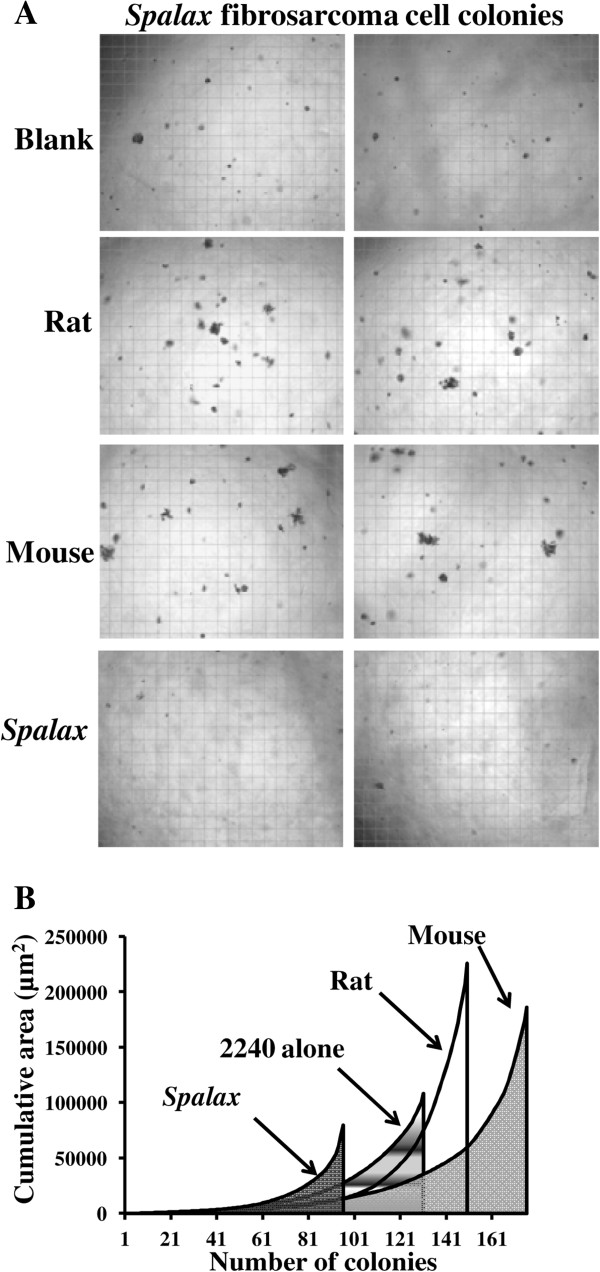
**Effect of *Spalax, *rat and mouse fibroblasts on *Spalax*-derived fibrosarcoma cells colony formation. (A) ***Sp*FS2240 Cancer cells were grown in soft agar on top of monolayers of mouse, rat and *Spalax* fibroblasts. After three weeks, colonies were counted. At least 10 fields were recorded for each observation. Two representative images demonstrating effects of different fibroblasts on colony-formation are shown (×40). **(B)** Colony numbers and cumulative total colony area (μm^2^) from five fields were calculated to demonstrate the effects of the fibroblasts monolayer on the cancer cell colony formation and growth.

Importantly, *Spalax* normal fibroblasts suppressed growth and colony formation of the homologous tumor, *Spalax-*derived fibrosarcoma (*Sp*FS2240) (Figure 11). In contrast, both rat and mouse normal fibroblasts stimulated growth of *Spalax* tumor cells in soft agar (Figure 11A). Integrating the number of colonies and their total occupied area, calculated from five independent fields, revealed a 36% reduction when *Sp*FS2240 were grown above a *Spalax* fibroblast monolayer compared to blank plates (Figure 11B, 2240 alone). In contrast, mouse and rat fibroblasts enhanced colony formation by factors of 1.7 and 2.1, respectively, compared to the blank plates (Figure 11B).

## Discussion

Notwithstanding the importance of laboratory mice in comprehension of carcinogenesis mechanisms, this cancer-prone model organism failed to provide satisfactory knowledge of cancer preventive mechanisms and treatment strategies in humans. (http://www.safermedicines.org/quotes/cancer.shtml). Therefore, elucidating mechanisms employed by a wild, non-inbred mammal that is naturally cancer-resistant raises promising opportunities.

*Spalax* has been thoroughly investigated at the Institute of Evolution of Haifa University as a model for adaptation to environmental hypoxia (for example, [1,23]). During the last decade a growing number of genes involved in hypoxic response have been studied and exhibited *Spalax*-specific features [4,11]. Directly related to this study on cancer-resistance of *Spalax* are the tumor suppressor *p53* favoring cell cycle arrest over apoptosis [24] and a unique *Spalax* heparanase splice variant that was shown to significantly decrease tumor size and metastatic activity compared to native heparanase [9]. Furthermore, despite the extremely long lifespan of *Spalax* (>20 years), even after studying thousands of individuals for decades, we have never observed animals that developed spontaneous tumors, nor show any aging-related phenotypic changes.

### *In vivo* studies of carcinogen-induced tumor

We report here that *Spalax* is resistant to two-stage DMBA/TPA, and 3-MCA carcinogen treatments. DMBA/TPA is commonly used to study malignant transformation, resembling formation of human squamous cell carcinoma [25]. A single dose of DMBA induced substantial oxidative stress [26], and when followed by repetitive application of TPA led to persistent inflammation supporting tumorigenesis [17]. In the present study, mice treated with DMBA/TPA initially developed benign papillomas, which subsequently transformed to squamous cell carcinomas. In contrast, treatment of *Spalax* led to necrotic wounds, which completely healed with no signs of malignancy. The carcinogen 3-MCA is known to produce fibrosarcomas through persistent inflammation and reactive metabolites causing severe oxidative damage [27]. In our study, 100% of 3-MCA-injected mice and rats developed tumors at the injection site within two to three and four to six months, respectively. One year after 3MCA treatment no *Spalax* animals showed any pathological process. However, 2 out of 6 old individuals (from a total of 12 animals) developed benign fibrotic overgrowths after 14 and 16 months, respectively, and only one case of malignant transformation in a >10 year-old *Spalax* animal was recognized, 18 months after 3-MCA injection.

It is well established that oxidative stress drives tumor progression and metastasis [28]. Thus, the mechanisms that *Spalax* evolved to survive hypoxia might be related to resistance to induced or spontaneous cancers. *Spalax* have recently been shown to have higher levels of reactive oxygen species (ROS) processing enzymes compared to hypoxia-intolerant rodents [4]. Nrf2, a transcription factor critical for defense against oxidative stress, has a unique structure in *Spalax.* Whereas it is highly conserved among most mammals [4], *Spalax* Nrf2 carries 27 specific amino acid replacements, 6 within the Neh6-domain, which is critical for stabilizing the protein under ambient oxidative stress and for its transcriptional activity [29]. Studies performed on Nrf2^−/−^ mice have shown the essential role of Nrf2 for detoxification of DMBA metabolites and protection against DMBA-induced carcinogenesis [30]. Unraveling the molecular mechanisms resulting in the healing of *Spalax* skin and inhibition of progression to tumor formation is the goal of our ongoing research. Hence, we have just initiated a comprehensive repetition of DMBA/TPA treatment where we will have a representative sample of animals from different stages following the application of this carcinogen in order to answer this question through quantification of apoptosis and senescence of *Spalax* skin and muscle tissue at the area of the carcinogen application. Furthermore, considering the high tolerance of *Spalax* to oxidative stress and the fact that DMBA is metabolized among others into ROS that cause oxidative DNA damage in the skin [31], the above experiment will allow us to compare the ROS levels upon DMBA application in *Spalax* and mice.

Another antioxidant enzyme, heme oxygenase-1 (HO-1), was shown to be elevated in *Spalax* tissues, and further increased under hypoxia [4,11]. HO-1 is involved in the degradation and catabolism of heme and supports synthesis of ferritin, an iron storage protein, thus preventing oxidative damage caused by free heme and ROS [32]. Indeed, most *Spalax* individuals, showing no external lesions following exposure to carcinogens, have probably resolved the initial inflammatory insult without excessive fibroplasias which can be attributed to more efficient anti-oxidation mechanisms. The benign fibroblastic proliferations observed in two 3-MCA-treated *Spalax* animals after 14 and 16 months suggest that *Spalax* is able to effectively arrest cancerous transformation. Nonetheless, whether *Spalax* tissues are able to prevent conversion of the 3-MCA pro-carcinogen into an active carcinogen, overcome its effect, or to inhibit previously transformed cells, remains to be clarified in future studies.

### *In vitro* studies of *Spalax* cancer resistance

Tumor growth and invasion are dependent on growth factors and cytokines produced by stromal cells [33]. Normal stroma contains a relatively small number of fibroblasts associated with ECM. However, during wound healing, fibrosis or malignant transformations, stromal fibroblasts proliferate, intensively produce growth factors and cytokines, express α-smooth muscle actin and become cancer-associated fibroblasts (CAFs) [12,34,35]. CAFs are removed from the granulation tissue rapidly after healing, though in cancer stroma they persist, contributing to epithelial mesenchymal transition of cancer cells. The later phenomenon is important for cancer progression and is mediated, at least in part, by metalloproteinases secretion and ROS generation [36,37]. Although the reports published to date have been mainly addressed to a cancer-promoting role of stromal fibroblasts, evidence suggests that normal stroma and normal fibroblasts could impede tumorigenesis [14-16,38,39]. Early studies [15] demonstrated that normal dermal fibroblasts suppressed development of malignant phenotypes of RAS-transformed keratinocytes when grafted into animals. Similarly, normal fibroblasts were able to retard melanomagenesis in its early stages [38]. Inhibition of growth and induction of differentiation were found in breast cancer pre-neoplastic MCF10-AT1-EIII8 cells when co-cultivated with normal fibroblasts, even in the presence of estrogen [39]. It is still unclear what events in the stroma, along with its interaction with precancerous cells, lead to a transition of the stromal function from cancer-protective to cancer-promoting, or, as in the present case of *Spalax*, what are the molecular mechanisms that *Spalax* evolved to escape cancerous transformation and to develop anti-cancer ability.

In a recent study [40], cancer resistance in *Spalax* was discussed. It was suggested that pro-growth signals originating from the fetal bovine serum, routinely added to culture medium, are conceived as cancerous transformation-like stimuli, driving *Spalax* fibroblast necrotic death, triggered through release of interferon-β (IFN-β). Nonetheless, in the same study, higher and earlier death rates were also shown in serum-reduced or serum-free media. Furthermore, the possibility that CM from “dying” cells may lack beneficial nutrients, or contain toxic metabolites, or other factors beyond IFN-β, was not addressed. Additionally, measurements of IFN-β in *Spalax* CM were performed indirectly using human cell lines [40]. The first, VSV (Vesicular Stomatitis Virus)-GFP (encoding a Green Fluorescent Protein) gene assay, measures IFN-β expression levels by VSV-GFP reporter assay. In this assay, HT1080 cell line (human fibrosarcoma cells) had been incubated with *Spalax* CM, and then infected with a GFP-encoding VSV. The level of IFN-β in the media corresponds to the reduction in the number of GFP positive human HT1080 cells. In the second assay, IFN-β release by “dying” *Spalax* cells is determined by HEK (Human Embryo Kidney cells)-Blue cells assay. In this assay the induction of β-gal reporter in human EK cells under IFN-β-inducible promoter is measured. Both assays use human cells for indirectly measuring *Spalax* IFN-β, which is inconsistent with the authors’ declaration that human cells are nonresponsive to *Spalax* CM stimuli possibly due to species divergence of IFN-β [40]. Likewise, no proof was given that the ability to kill “dying” fibroblasts is unique to *Spalax’*s CM, for example, by trying to compare the fate of the cells when grown with CM of the other species tested in the study, namely, mice or human. Additionally, the method used in this study for declaring necrotic death is based on the Annexin V/propidium iodide assay [40]. Briefly, floating and adherent cells were harvested, stained with Annexin-V and propidium iodide, and analyzed by flow cytometery. The known disadvantage of this method is that it cannot conclusively prove that cell death is solely the result of necrosis, nor eliminate the possibility of apoptotic mechanisms. Also, the authors have not provided evidence for interrelations between their three declared observations (transformation-like stimuli, necrotic death and release of IFN-β). Overall, it is our impression that the above mentioned study [40] does not provide direct evidence to *Spalax* cancer resistance, certainly not its anti-tumor properties. Alternatively, we show here that viable, proliferating *Spalax* fibroblasts, from adult and newborn animals, inhibit growth of cancer cells derived from different tissues and species, most importantly human, but do not affect non-cancerous cells, including those of *Spalax* (Figure 7F), thereby highlighting a strategy used by *Spalax* to identify and target malignancies. This unique interaction is further strengthened by the observation that the growth of cancer cells is regained once the immediate interaction with *Spalax* cells is terminated (Figure 7D). Importantly, no inhibitory effect on cancer cell growth was found when fibroblasts from above-ground species (rat, mice and *Acomys*) were tested.

Recently, several studies investigated the unique cancer-resistance properties of the naked mole rat (*Heterocephalus glaber*)*,* another subterranean, long-lived, rodent species. The most recent study suggested a connection between a high viscosity of media conditioned by *Heterocephalus* fibroblast cells due to exceptional secretion of high-molecular mass hyaluronan (HMM-HA) [41], which was suggested to mediate what was named by the authors “early contact inhibition”, previously described by the same group as an anticancer mechanism in *Heterocephalus* cells*,* and was initially ascribed to p16(Ink4a) and p27(Kip1) activity [42]. In the same paper [41], it is reported that HMM-HA was detected also in *Spalax* fibroblasts even in higher levels compared to *Heterocephalus* fibroblasts, though no experiments were carried out to clarify its role in *Spalax* fibroblasts. Nevertheless, this may explain the prevalent high viscosity of the medium of cultured *Spalax* fibroblasts we noticed, though we find that it does not prevent *Spalax* cells from reaching confluence or influences their anti-cancer properties. Furthermore, CM from *Spalax* with apparent normal viscosity was also able to inhibit cancer cells proliferation (ongoing study). In light of the fact that hyaluronan-cancer cell interactions were shown to promote, and not inhibit, cancer invasion [43], the correlation between HMM-HA, the potential of cells to reach confluence and the resistance to oncogenic transformation or anti-cancer activity, requires further direct experimental support, especially in the case of our model organism, the *Spalax*. Another study endorsed *Heterocephalus* cells’ cancer-resistance to rapid cell crisis following oncogenic transformation, which is characterized by abnormal chromatin material and nuclei, leading to a failure to successfully complete cell division, hence the inability of the cells to progress into malignancy [44]. These observations are somewhat similar to our findings of fragmented and deformed nuclei and chromatin condensation (Figure 8), disturbed cell division and proliferation (Figure 9E) of human cancer cells, as well as the 3MCA-induced *Spalax* and mice fibrosarcoma cell line, upon their interaction with *Spalax* fibroblasts. In view of the similar ability of *Heterocephalus* fibroblasts to kill cancer cells (Figure 6), and as the efficiency of experimental oncogenic transduction of cells is never 100%, it is possible that the *Heterocephalus* cells that escaped malignant transformation killed the oncogenic-transduced ones.

Our findings demonstrated that *Spalax* fibroblasts or their CM target human cancer cells growth machinery, triggering programmed cancer cell death (Figures 4, 5, 8 and 9). Following co-culture with *Spalax* fibroblasts or their CM, cancer cells (Hep3B, HepG2 and MCF7) undergo morphological changes typical of apoptosis [45]: swelling, rounding, detachment, shrinkage and floating. Moreover, nuclear condensation and abnormal mitochondrial fission as well as accumulation of cells in sub-G1 (Figures 8 and 9) also suggest apoptotic modes of cancer cell death. BrdU incorporation, reflecting cell proliferation, confirmed that *Spalax* CM contains anti-proliferative factors, inhibiting cell division in a time-dependent pattern. We further showed that the effect of *Spalax* CM on cancer cells is transient and reversible. That is, replacing the CM with regular fresh medium leads to recovery of those cancer cells that had not been affected by the CM. Last but not least, *Spalax* fibroblasts presumably impair the aggressive behavior of tumor cells: the invasive phenotype of highly metastatic MDA-MB-231 breast carcinoma cells was markedly reduced (Figure 10). Noteworthy, the ability to form colonies in soft agar by 3MCA-induced, *Spalax*-derived fibrosarcoma was significantly suppressed by homologous fibroblasts, whereas heterologous fibroblasts (rat and mouse) increased tumor formation (Figure 11). *Spalax* fibroblasts also inhibited colony formation in soft agar by 3MCA-induced, mouse-derived fibrosarcoma.

In order to strengthen our findings of *Spalax* cells’ anti-cancer activity, compared to cells from laboratory, in-bred, aboveground mice and rats, we decided to follow the cancer activity pattern of two other, wild, out-bred, species. Hence, fibroblast cells were propagated from the aboveground, wild, short-lived rodent *Acomys,* and the subterranean, wild, long-lived *Heterocephalus.* We have shown here that, similar to *Spalax* cells, *Heterocephalus* fibroblasts restrict growth and effectively kill cancer cells, while *Acomys* cells behave similarly to rat and mice, that is, have no anti-cancer activity (Figure 6). We may assume that this anti-cancer ability might be shared by species living under extreme conditions and adapted to stress, such as hypoxia, which is directly related to cancer initiation and progression. It would be interesting to investigate this phenomenon in other hypoxia-tolerant species, such as other subterranean, high altitude and diving mammals.

Previous studies showed that key hypoxia-regulatory genes in stromal fibroblasts, such as *HIF1-α* and *VEGF*, negatively influence tumorigenesis [46]. HIF1-α is a known tumor-promoting transcription factor in most malignancies [47]; however, its expression in tumor stromal fibroblasts could suppress cancer cell growth [46]. We have previously shown that *HIF1-α*[23] as well as ROS-scavenging enzymes [4] are constitutively highly expressed in *Spalax*. Similar to our explanations of the failure to induce cancer *in vivo* in live *Spalax* animals, *in vitro* studies, using fibroblast cells, demonstrated a significant role in adaptive response to oxidative stress, at least in part, via expression of HO-1 [48]. High levels of mitochondrial ROS produced by cancer cells were shown to drive tumor development via remodeling of the stromal environment and enhancing invasion. Recently, the roles of ROS produced by fibroblasts in their trans-differentiation to myofibroblasts and in cancer cell invasiveness were reported [37]. ROS-generating CM of mutated fibroblasts promoted metastasis of A375 melanoma through the increasing of ROS and HIF1-α stabilization in melanoma cells. However, when N-acetyl cysteine, a ROS scavenger, was added to the system, HIF1-α accumulation and melanoma cell invasion were inhibited [37].

Adaptive tolerance to hypoxia stress in *Spalax,* both *in vivo* and *in vitro,* may grant the unique resistance to cancer through strong antioxidant mechanisms, among others (for example, as mentioned here, the unique activity of its p53 [8] and heparanse [9]), that quench ROS before they spread and damage DNA and other macromolecules, thus providing cellular homeostasis and cancer protection. As such, they are a milestone in our efforts in understanding the mechanisms by which the long-lived, hypoxia-tolerant *Spalax* hinders cancer initiation and progression.

Collectively, we have shown here an outstanding cancer resistance of the whole, live *Spalax*, and not just in cultured cells, and anticancer activity of *Spalax* cells on human cancer cells, and not just resisting transformation of its own healthy cells. This phenomenon extensively described here using different methodologies on cells from different ages of *Spalax,* together with our initial observation of a similar ability of cells originated from another subterranean, long-lived, hypoxia- and cancer-resistant animal, the *Heterocephalus,* highlight the importance to adopt such animal models with exceptional genetic-embedded tolerance to environmental stress, in cancer research.

Our ongoing research is focused on identifying the factors secreted by *Spalax* cells, and their selective interaction with cancer cells to suppress tumorigenesis. Our first step to exploring the nature of the secreted factors was the heat-inactivation preliminary experiment presented here (Figure 7G). The heat treatment of *Spalax* CM caused only partial loss of the anti-cancer activity of *Spalax*-generated CM. Although not conclusive, this may indicate the involvement of protein factors in the observed phenomenon. We are also studying the signaling mechanisms and death receptors whose activation triggers cancer cell death. These studies will hopefully contribute to the identification of new anti-cancer mechanisms and future tumor preventive or therapeutic strategies. To our knowledge, the present study demonstrates, for the first time, *Spalax* tolerance to chemically induced carcinogenesis along with direct anticancer effect of *Spalax* fibroblasts on human cancer cells.

## Conclusions

During 50 years of studies, with thousands of animals that crossed our Animal House, we have never observed spontaneous tumors in *Spalax*. Similar phenomenon was observed also in another subterranean long-lived rodent, *Heterocephalus.* Based on this observation, a few studies [41,42,44] have tried to explain cancer resistance in *Heterocephalus* through testing known molecular mechanisms of malignant transformation on healthy fibroblast cells. Though different molecular mechanisms were suggested, none of these studies dealt with anti-cancer properties of the whole, live animal *in vivo*, or on the direct interaction of its normal cells with cancer cells. Hence, our presentation is a pioneering, genuine, breakthrough study. We have tried to induce cancer in *Spalax* with chemical carcinogens that induced cancer in 100% of mice and rats, and the results allow us to state that *Spalax* is extremely resistant not only to spontaneous cancer but also to induced cancer. Furthermore, fibroblast cells from *Spalax* inhibit growth and kill cancer cells from various species and cell lines. This is exhibited in both the co-culture system or by exposure to conditioned media harvested from *Spalax* fibroblasts. Cancer cell death was accompanied by decreased cancer cell viability and proliferation, reduced colony formation in soft agar, disturbed cell cycle progression, chromatin condensation, nuclei deformation and mitochondrial fragmentation.

This phenomenon is prominent in *Spalax* fibroblasts, regardless of the animal age, from newborns a few days old to animals over 10 years old, as proved by the reversibility of cancer cells death, once their immediate interaction with *Spalax* fibroblasts is terminated. The anti-cancer activity of *Spalax* is specific to cancer cells and not to normal cells. It may be shared by other stress-adapted mammals as we initially showed here by co-culturing *Heterocephalus* fibroblasts with cancer cells that also leads to cancer cell death.

“*The classical mice model for cancer research has little predictive value and a negligible relation to that of human. Far more than anything else, the lack of good animal models has become the rate-limiting step in human cancer research*” (Prof. Robert Weinberg, MIT; *Newsweek, September 6th, 2008*). Therefore, it would be extremely useful to study naturally cancer-resistant animals, as models to find ways to prevent cancer before it occurs. Our results may lead to a breakthrough in the conservative paradigm of cancer research, completely dependent on laboratory, inbred rodents, and place *Spalax* as the ‘missing’, appropriate candidate model for such studies. We anticipate that the long lived, hypoxia- and cancer-tolerant *Spalax* will turn out to be a significant biological resource to biomedical research as a model organism for understanding cancer and its prevention.

## Methods

### Animals

All animal protocols were approved by the Institutional Ethics Committee.

Blind mole-rat (*Spalax),* rats (*Rattus norvegicus*) and C57BL/6 mice were subjected to DMBA/TPA or 3MCA treatments. For DMBA/TPA treatment, eight *Spalax* and six mice were used. For 3MCA treatment 12 *Spalax*, 6 mice and 6 rats were used. *Spalax* and *Acomys* were captured in the field and housed under ambient conditions in individual cages in the Animal Facility of the Institute of Evolution, University of Haifa. Noteworthy, *Spalax* do not undergo uniform acclimatization upon transfer from their natural habitat to a standardized laboratory environment but rather behave differentially according to their eco-geographic origins [49]. The C57BL/6 mice were purchased from Harlan Laboratories (Jerusalem, Israel). Rats were supplied by the Animal House of the Psychology Department of Haifa University. *Heterocephalus* was a gift from Tisch Family Zoological Gardens in Jerusalem. All animals were kept with free access to food and water at 21 to 23°C in a 12:12 light-dark cycle. All animals used for experiments were healthy. Animals were sacrificed with an inhalation anesthesia agent (isoflurane) overdose.

### DMBA/TPA treatment

Four *Spalax* individuals approximately 2 years old and four individuals over 10 years old; and six individuals of C57BL/6 mice, 3 to 4 months old, were used in the 7,12-Dimethylbenz(a)anthracene/12-*O*-tetradecanoylphorbol-13-acetate (DMBA/TPA) experiments. A single application of 200 μg of DMBA dissolved in 100 μl of acetone for mice, and 500 μg in 250 μl for *Spalax* was used. The solution was applied onto the shaved back skin of the animal. Three days after the initial DMBA dose, mice were treated with 30 μg of TPA (Sigma Aldrich, Inc.) dissolved in 100 μl of acetone, and *Spalax* with 60 μg of TPA dissolved in 250 μl of acetone. TPA was applied three times per week for two to three months, until all mice developed advanced cancer and were subsequently sacrificed. *Spalax* continued to be treated for an additional three months twice a week.

### 3-MCA carcinogen treatment

3-methylcholanthrene (3-MCA) has been commonly used for induction of tumors in rodents [50]. In this experimental system, mice and rats develop local fibrosarcomas in two to three and four to six months, respectively [20]. The recommended dose of 3MCA (Sigma Aldrich, Inc.) for treatment of mice is 200 μg dissolved in 200 μl of olive oil [20,51]. We calculated the amount applied to rats and *Spalax* according to their average weight. Hence, animals were treated with a single injection of 3MCA as follows: 200 μg/200 μl for mice; 1 mg/500 μl for *Spalax*; and 1.5 mg/500 μl for rats. Animals used in this experiment were: six approximately 2-year-old *Spalax* individuals; six 10-year old or older *Spalax* individuals; six 3- to 4- month-old mice; and six 3-month-old white rats. Animals were observed once a week until tumors could be palpated, and then two to three times a week. Animals were sacrificed; tissues were removed for cell isolation or fixation. For histological examination, the samples were fixed in 4% paraformaldehyde dissolved in PBS, dehydrated in increasing concentrations of ethanol, and embedded in paraffin. Five-micrometer sections were cut from paraffin blocks and routinely stained with hematoxylin and eosin for microscopic examination.

### Cell culture

Primary *Spalax*, mice, rat, *Acomys* and *Heterocephalus* fibroblasts were isolated from under arm skin and lungs as described [52], and grown in DMEM-F12 medium (Biological Industries, Beit Haemeq, Israel), supplemented with 15% fetal bovine serum (FBS). Human cancer cell lines Hep3B and HepG2 (hepatoma-derived), MCF7 and MDA-MB-231 (breast cancer cells) were obtained from ATCC, and were grown in RPMI (Hep3B, HepG2) and DMEM (MCF7, MDA-MB-231) supplemented with 10% FBS, L-glutamine, penicillin and streptomycin (Biological Industries). Cells were incubated in a humidified atmosphere of 5% CO_2_ and 95% ambient air at 37°C. *Spalax*-derived fibrosarcoma cells were isolated from a tumor developed after 3MCA injection. Tumor specimens were minced and treated with 1 mg/ml of collagenase (Sigma-Aldrich) under aseptic conditions to obtain a single-cell suspension, which was plated in culture dishes in DMEM-F12 medium supplemented with 15% FBS and penicillin-streptomycin-amphotericin B solution. Cells were serially cultured more than 40 times.

### Co-cultures of cancer cells and fibroblasts

Normal fibroblasts and human-derived cancer cells were co-plated in six-well plates in 2 ml of culture medium RPMI or DMEM/DMEM-F12 (1:1) supplemented with 15% FBS. Fibroblasts were plated first (5 × 10^4^), and cancer cells were added within 1 h (5 × 10^3^), with a 10:1 fibroblast-to-cancer cell ratio. In parallel, control cultures of cancer cells and fibroblasts were plated at the same number of cells. The medium was changed every three days. Fibroblast-cancer cell co-interactions were observed and photographed by using an inverted microscope (Optika XDS2, Italy).

### Generation of conditioned media

Normal fibroblasts or cancer cells (1 × 10^6^ cells) were plated in 10-cm tissue culture dishes and cultured in full medium containing 10% FBS for four days; thereafter, media were collected and cells were removed by centrifugation. The cell-free supernatants were then diluted with the appropriate fresh culture medium (1:1) and used for further experiments. Trypan-blue standard treatment followed by cell count using a Countess® automatic cell counter (Life Technologies), and PrestoBlue® dye reagent (Invitrogen) (as described in [53]) were used to investigate the viability of cancer cells exposed to CM of normal fibroblasts from different species. For BrdU incorporation assay, Cell Proliferation ELISA (Exalpha) was used following the manufacturer’s instruction.

### Soft agar colony formation assay

Colony formation assay was performed as described [54]. In brief, 2 × 10^5^ fibroblasts were seeded in 35-mm culture dishes and cultured for two to three days to reach confluence. After washing with PBS, 1 ml of 0.5% agar in DMEM-F12 containing 2% FBS was added on top of the fibroblasts to form a base layer. After the agar was solidified, 5 × 10^5^ cancer cells were suspended in 1 ml of 0.35% agar in DMEM containing 5% FBS and then added into the dish to form a cancer cell layer. Visible colonies were studied and photographed under phase-contrast microscope. Finally, colonies larger than 50 μm were counted. At least 10 fields on each plate duplicate were used for counting at a magnification of × 40. For total colony area, five fields were analyzed using ImageJ software [55].

### Cell cycle analysis

The cell cycle distribution was assessed by flow cytometry of propidium iodide (PI)-stained nuclei as described previously [56]. In brief, following incubation, cells were harvested by trypsin, combined with medium containing floating cells, washed with PBS and stained with hypotonic PI solution (PI 50 μg/ml in 0.1% sodium citrate and 0.1% Triton X-100). The PI fluorescence of individual nuclei was recorded by FACSaria (Becton Dickinson, NJ, USA). A total of 10,000 events were acquired and corrected for debris and aggregate population.

### Nuclear and mitochondrial staining

Cells grown on cover slips were stained with MitoTracker®Red CM-XRos (Life Technologies) at 37°C in a humidified 5% CO_2_ atmosphere for 15 minutes and fixed with 3.7% formaldehyde in culture medium for another 15 minutes at 37°C. After washing with PBS twice, nuclei were counterstained with DAPI. Images were acquired with a fluorescence microscope.

### Transmission electron microscopy

For transmission electron microscopy, specimens were fixed in 2.5% glutaraldehyde in 0.1 M sodium cacodylate buffer (pH 7.2), postfixed with 2% OsO_4_, dehydrated in ethanol series and embedded in epoxy resin. Semi-thin sections were stained with 1% Toluidine Blue. Ultrathin sections (60 nm) were cut with a diamond knife, placed on 300-mesh copper grids, stained with 1% uranyl acetate, and viewed and photographed with a transmission electron microscope (Technai T12, FEI).

## Abbreviations

3-MCA: 3-Methylcholanthrene; BrdU: Bromodeoxyuridine; CAF: Cancer-associated fibroblasts; CM: Conditioned media; DAPI: 4’,6-Diamidino-2-phenylindole; DMBA/TPA: 7,12-Dimethylbenz(a) anthracene/12-*O*-tetradecanoylphorbol-13-acetate; DMEM: Dulbecco’s modified Eagle medium; ECM: Extracellular matrix; FBS: Fetal bovine serum; GFP: Green fluorescent protein; HEK: Human embryo kidney cells assay; HIF1-α: Hypoxia-inducible factor 1 alpha; HO-1: Hemoxygenase-1; IFNβ: Interferon beta; PBS: Phosphate-buffered saline; PI: Propidium iodide; ROS: Reactive oxygen species; TC: Tumor cells; VEGF: Vascular endothelial growth factor; VSV: Vesicular stomatitis virus.

## Competing interests

The authors declare that they have no competing interests.

## Authors’ contributions

AA and IS are joint senior authors. AA, IS and IM share major and equal contributions to this study. They conceived the study, designed the experiments, performed and analyzed data, and wrote the paper. AA and NS planned and executed the live animals’ *in vivo* experiments. IS and IM had a key role in the development and presentation of the *in vitro* studies. MH carried out the histopathological analysis and interpretation. IM and TCI implemented and analyzed the ultrastructure experiment by electron microscopy. MB and AM were responsible for the functional genomics studies, which serve as a partial basis of this report; they have been involved in the experimental planning, data analysis and writing of the manuscript. All authors read and approved the final manuscript.
